# Supplementary material for: Patterns of Intron Gain and Loss in Fungi
Source: PLoS Biol. 2004 Nov 30;2(12):e422. doi: 10.1371/journal.pbio.0020422 (PMC532390; doi:10.1371/journal.pbio.0020422)
Supplement: Table S1 — Also available at http://genes.mit.edu/NielsenEtAl/. (4.3 MB ZIP). [file pbio.0020422.st001.zip › NielsenEtAl/html/269.html]

AN0293.1.NCU00954.1.MG01649.1.FG00587.1


```
 CLUSTAL W (1.82) Multiple Sequence Alignments - Introns Inserted


Sequence 1: MG01649.1	424 aa
Sequence 2: FG00587.1	400 aa
Sequence 3: NCU00954.1	454 aa
Sequence 4: AN0293.1	425 aa
Alignment Length: 515 aa
Number Identitical Residues: 199 aa
Alignment Score (without introns) 9165


MG01649.1 	MSELQKRWAKARVDPMASTPQ------------FDEADEQQAPTYQTQQQPKDYLEETGT
NCU00954.1	MSELQKQWAKARLGSQALGQPPAEMPITTTMPEFNEADEDKE---------------MAG
FG00587.1 	MSDLQRTWAKAKFG--ASNDP------------FDEPQEEQG---------------QDV
AN0293.1  	MSDLQKSFAKSKLAKLPPEPP--------------------------------------P
          	**:**: :**::.   .                                           

MG01649.1 	VPELPELPEDDSSSASSAS---STGTIVPSVQQNLFARPQG2LSR~GRTLEPVPWTTYFE
NCU00954.1	PSPGGFPFDDDSSSASSVS---STGTVIPSPGRALFARPQG2FAS~-RSMDPIPWTTYFE
FG00587.1 	LPELPEPVDDDSSSASSASSVSSTGTIIPSPNQKLFARPQG2VAR~GRTLEQIPWTTYFE
AN0293.1  	IPESVADEDDDSGS--------STETVTPSPVKQLFARPGG~TRG2-----------FFD
          	 .      :***.*        ** *: **  : ***** *                :*:

MG01649.1 	RELFLEVETEGSR----------ATNTFHAYLTSPVGKGPLFVTHHGAGSSGLSFAIVGA
NCU00954.1	RELFLKEEAGPDSGSRTSNKNKPTSITYHAYLTSPVGKGPLFVTHHGAGSSGLSFAVLSS
FG00587.1 	REVSLKSEQD-------------PEVIYHAYLTSPVGKGPLFVMHHGAGSSGLSFAVVAS
AN0293.1  	QELYLQRRVN------------DLDIVHHVYLTSPTNSGPLFVMHHGAGSSGLSFANCAA
          	:*: *: .                   .*.*****...***** ************  .:

MG01649.1 	EVRKLNPSAGILSLDARGHGSTTTS----------------SPGVDLSLETLAADLLGVI
NCU00954.1	EIRKRLPNAGILSLDARGHGSTTVTAASSVPGEGDETTGQAPPPLDLSLSTLASDLFTVI
FG00587.1 	QIRKRISTAGILALDCRGHGSTYAPE---------------DKAFDMRLDTLSSDLYNVV
AN0293.1  	EIRKILPNAGILSADARDHGSTSVKRASE-----DGEADPETARLDLSLDTLNQDLLFVI
          	::**  ..****: *.*.**** .  ::.     .  :.     .*: *.**  **  *:

MG01649.1 	HATKDKMGWQALPPMVLVGHSLGGAVVTHLAGLRRLDDSVLLGYAVLDVVEG--------
NCU00954.1	QLTRTAMHWPELPPIILVGHSLGGAVVTELAKSYRLGPSLLG-YAVLDVVEG--------
FG00587.1 	QLTKTEMSWPEMPPIVLVGHSLGGAVVTDLAKSGKLGTSVLG-YAVLDVVEG--------
AN0293.1  	RETQAKMGWETLPDIVLVGHSLGGAVITDVAKKGELGGKLLA-YAVLDVVEGGFQPISGP
          	: *:  * *  :* ::**********:*.:*   .*. .:*  *********. .. :..

MG01649.1 	----------SAMDALQSMQTYLSTRPTGFASVPAAIDWH~VRSRTIRNSVSARTSVPAL
NCU00954.1	----------CAMDALQSMQTYLSTRPQGFASLKDGIDWH~VRSRTIRNSTSARTSVPGL
FG00587.1 	----------SAIDALQSMHTYLSTRPLGFATLQAGIEWH~IRSRTIRNSISARTSVPAL
AN0293.1  	GRGRSCRTLGSAMDALQSMETYLSTRPSRFPSLPSGIEWH2TRSRTIRNRTSARVSVPSL
          	. . :. : ..*:******.*******  *.::  .*:**  *******  ***.***.*

MG01649.1 	LTHCTDGA---------DEGAGGGNSRPWRWRTDLAATQPFWEGWFVGLSKKFLTGRGGK
NCU00954.1	LAPVEELQRPELQQLPRGVAGTQGTAKPWRWKTDLAATQPFWEDWFVGLSKKFLEARGGK
FG00587.1 	LVFNEN-----------DD-----PTRPWRWRTNLGATQPYWEDWFVGLSKKFLEARGGK
AN0293.1  	LYHEDVPK---------------DPSKPWVWRTNLAETKPFWEGWFVGLSRKFLEARGGK
          	*                      . ::** *:*:*. *:*:**.******:*** .****

MG01649.1 	MLLVAGTDRLDTELTIGQMQG1KYALQVFPEAGHFIHEDLPEKTAIALVDFHRRNDRSVM
NCU00954.1	MLLLAGTDRLDTELTIGQMQG1KYALQVFPEAGHFIHEDLPEKTAIALVDFHRRNDRSQL
FG00587.1 	MLLLAGTDRLDTELTIGQMQG1KYALQVFPEAGHFIHEDLPEKTAVSLVDFFRRNDRTAL
AN0293.1  	LLLLAGTDRLDKELMIGQMQG1KYQLQVFPDAGHFIQEDQPARTAQILVDFYKRNDRSAL
          	:**:*******.** ****** ** *****:*****:** * :**  ****.:****: :

MG01649.1 	VLPPKVADLIA-------QGKRV----------------
NCU00954.1	VLPPKVSDLLA-------QGKKV----------------
FG00587.1 	VLPPKVSDLIK-------QGKRV----------------
AN0293.1  	VLPPKVADMQASAAMKKGAGAGVPLGKAEGGTTGSFKRS
          	******:*:  ::: ... *  *. ..:...::.: . :
```
